# Crystal structure of human CRMP-4: correction of intensities for lattice-translocation disorder

**DOI:** 10.1107/S1399004714006634

**Published:** 2014-05-30

**Authors:** Rajesh Ponnusamy, Andrey A. Lebedev, Steffen Pahlow, Bernhard Lohkamp

**Affiliations:** a Instituto de Technologia Química e Biológica, Universidade Nova de Lisboa, Avenida da República, EAN, 2781-901 Oeiras, Portugal; b Research Complex at Harwell, STFC Rutherford Appleton Laboratory, Didcot OX11 0FA, England; c Biozentrum Klein Flottbek, University of Hamburg, Ohnhorststrasse 18, 22609 Hamburg, Germany; d Department of Medical Biochemistry and Biophysics, Karolinska Institutet, Tomtebodavägen 6, 4tr, 17177 Stockholm, Sweden

**Keywords:** CRMP-4, lattice-translocation disorder

## Abstract

Collapsin response mediator proteins (CRMPs) are cytosolic phosphoproteins that are mainly involved in neuronal cell development. In humans, the CRMP family comprises five members. Here, crystal structures of human CRMP-4 in a truncated and a full-length version are presented. The latter was determined from two types of crystals, which were either twinned or partially disordered. The crystal disorder was coupled with translational NCS in ordered domains and manifested itself with a rather sophisticated modulation of intensities. The data were demodulated using either the two-lattice treatment of lattice-translocation effects or a novel method in which demodulation was achieved by independent scaling of several groups of intensities. This iterative protocol does not rely on any particular parameterization of the modulation coefficients, but uses the current refined structure as a reference. The best results in terms of *R* factors and map correlation coefficients were obtained using this new method. The determined structures of CRMP-4 are similar to those of other CRMPs. Structural comparison allowed the confirmation of known residues, as well as the identification of new residues, that are important for the homo- and hetero-oligomerization of these proteins, which are critical to nerve-cell development. The structures provide further insight into the effects of medically relevant mutations of the *DPYSL-3* gene encoding CRMP-4 and the putative enzymatic activities of CRMPs.

## Abstract 2

Crystals of human CRMP-4 showed severe lattice-translocation disorder. Intensities were demodulated using the so-called lattice-alignment method and a new more general method with simplified parameterization, and the structure is presented.

## 1. Introduction   

Collapsin response mediator proteins (CRMPs) are cytosolic phosphoproteins that are highly expressed in the developing nervous system (Charrier *et al.*, 2003 ▶; Schmidt & Strittmatter, 2007 ▶). The CRMP protein family comprises five members. CRMP-4 shares 72–79% sequence identity with CRMP-1, CRMP-2 and CRMP-3 and 52% with CRMP-5, and also 58% with the enzyme dihydropyrimidinase (DHPase). Despite the high similarity of CRMPs to DHPase, none of them shows DHPase activity (Ponnusamy & Lohkamp, 2013 ▶; Wang & Strittmatter, 1997 ▶). On the other hand, CRMP-3 (but not CRMP-4) has been shown to exhibit histone deacetylase activity (Hou *et al.*, 2013 ▶). Several CRMPs, CRMP-1, CRMP-2 and CRMP-4, occur in two alternatively spliced isoforms. The longer, less ubiquitously expressed form (L-CRMP or b) contains an extra N-terminal domain with unknown structure and function compared with the short form (S-CRMP or a) (Quinn *et al.*, 2003 ▶; Yuasa-Kawada *et al.*, 2003 ▶). CRMPs have been shown to form homotetramers as well as heterotetramers *in vivo* (Wang & Strittmatter, 1997 ▶). Compared with DHPases, CRMPs have an extended C-terminus, which is positively charged, contains phosphorylation sites and is highly susceptible to proteolysis (Deo *et al.*, 2004 ▶; see below). However, this C-terminal region is cleaved *in vivo* by the calcium-activated protease calpain to produce 55–58 kDa products in response to neuronal cell injury and neurite damage (Jiang *et al.*, 2007 ▶; Zhang *et al.*, 2007 ▶). The effect of the C-terminal truncation correlates with the presence of an inhibiting factor for post-injury neurite regeneration, but how this relates to the structure and function of CRMPs is unknown. Knowledge of the structure and oligomerization properties of all CRMP family members is thus required to understand the unique role of these proteins.

CRMP-4 is encoded by the gene *DPYSL3* (dihydro­pyrimidinase-like protein 3). Like the other CRMPs, it is involved in neuronal development and binds to tubulin dimers and microtubules. CRMP-4 has also been shown to increase neurite extension and branching by interacting with the SH3A (Src homology 3A) domain of intersectin (Quinn *et al.*, 2003 ▶). CRMP-4 knockout mice show increased proximal bifurcation in the CA1 hippocampus, while CRMP-4 together with CRMP-2 synergistically regulate dendritic development (Niisato *et al.*, 2012 ▶, 2013 ▶). The differential phosphorylation status of CRMPs is highly regulated during neuronal development by inhibiting CRMP interaction with various cyto­skeletal proteins (Yamashita & Goshima, 2012 ▶). Similar to the other members of the CRMP family, three residues (Thr509, Thr514 and Ser518) at the C-terminus of CRMP-4 are phosphorylated by GSK-3β (Cole *et al.*, 2004 ▶). However, CRMP-4 is phosphorylated at Ser522 by DYRK2, unlike other CRMPs, which are phosphorylated by Cdk5 as a priming kinase for phosphorylation by GSK-3β (Cole *et al.*, 2006 ▶). CRMP-4 binds to RhoA, an important regulator of cell-cycle progression and cytokinesis, but phosphorylation of CRMP-4b hampers this binding, resulting in inhibition of axon regeneration (Alabed *et al.*, 2007 ▶, 2010 ▶). Furthermore, CRMP-4 promotes F-actin bundling and regulates chromosomal alignment (Ong Tone *et al.*, 2010 ▶; Rosslenbroich *et al.*, 2005 ▶). These observations suggest that CRMP-4 not only plays a role in nerve-cell development, but is also involved in the regulation of microtubule dynamics during mitosis. This may explain why CRMP-4 is linked to various cancers: its expression is associated with liver metastasis, poor survival in pancreatic cancer (Hiroshima *et al.*, 2013 ▶) and neuroblastoma (Tan *et al.*, 2013 ▶), whereas in prostate cancer CRMP-4 functions as a metastasis suppressor (Gao *et al.*, 2010 ▶). Antibodies against CRMP-4 have been related to limbic encephalitis and thymoma (Knudsen *et al.*, 2007 ▶). Additionally, CRMP-4 has been introduced as a therapeutic target in ischaemic stroke (Sugunan *et al.*, 2013 ▶). A CRMP-4 variant was identified in French amyotrophic lateral sclerosis (ALS) patients that appears to shorten motor neuron survival through a detrimental effect on axonal growth (Blasco *et al.*, 2013 ▶).

Among the different types of crystal pathologies, twinning is the most familiar to macromolecular crystallographers (Hahn & Klapper, 2013 ▶), particularly twinning by (pseudo)mero­hedry. Another type of crystal abnormality is partial disorder (see, for example, Pletnev *et al.*, 2009 ▶); attempts to use such crystals for structure determination are much less frequently undertaken than for twinned crystals. In partially disordered crystals, the long-range translational symmetry is missing in one or more directions, resulting in poorer diffraction and elongated and streaky spots in the diffraction images. A polysynthetic twin with small twin domains is an example of a partially disordered structure in which adjacent domains have different orientations. In this work, both abnormalities, twinning and partial disorder, were encountered in two different crystal forms of full-length CRMP-4.

The term lattice-translocation disorder (Wang, Kamtekar *et al.*, 2005 ▶) is frequently used in macromolecular crystallo­graphy to denote partially disordered crystals in which the ordered domains have the same orientations but are displaced from the positions where they would have formed a single crystal. The translocation disorder in macromolecular crystals was first observed in 1954 in crystals of imidazole methaemoglobin (Howells & Perutz, 1954 ▶). It is usually characterized by a diffraction pattern which contains streaky diffraction spots in addition to well defined Bragg reflections. Furthermore, non-origin Patterson peaks are observed resulting from an interference term between neighbouring crystal domains. These peaks correspond to the cross-vectors between molecules that are located in different domains but have the same orientation. In this sense, such Patterson peaks have the same nature as the peaks arising from translational noncrystallographic symmetry (tNCS). However, Patterson peaks resulting from lattice-translocation defects are usually located too close to the origin, so they cannot be reconciled with any sensible packing of molecules in the crystal structure.

In special cases, where the translation relating adjacent domains equals the translation relating equivalent (but not identical) crystallographic origins in ordered domains, multi-model refinement can be performed. Overlapping molecules with adjusted occupancy form a synthetic crystal structure that is refined by the usual techniques (Wang, Kamtekar *et al.*, 2005 ▶, Zhu *et al.*, 2008 ▶). However, owing to overlapping of the molecules in alternative positions the resulting electron density may be difficult to interpret, and this method can only be used in special cases. In a more general approach, the two lattices related by translocation are computationally aligned, in the sense that the coherence term is removed from observed intensities using a statistical model including two parameters: the translocation vector and the relative amplitude of the interference term (Wang, Rho *et al.*, 2005 ▶; Hare *et al.*, 2009 ▶).

Here, we present the crystal structure of human CRMP-4 solved from different protein variants in different space groups. One crystal form was twinned and another showed severe lattice-translocation disorder. For the latter, the intensities were corrected using several procedures including a novel method, DIGS (demodulation of intensities by grouping and scaling), that improved the refinement statistics and the interpretability of the maps.

## 2. Materials and methods   

### 2.1. Cloning and expression   

The regions coding for CRMP-4 (residues 1–570; NP_001378.1) and a truncated version of CRMP-4 (CRMP-4 ΔC, residues 13–490) were amplified by PCR from a cDNA fragment containing the *DPYSL3* gene. For PCR, the following primers were used: 5′-*TAC TTC CAA TCC* ATG TCC TAC CAA GGC AAG AAG-3′ and 5′-*TAT CCA CCT TTA CTG TTA* ACT CAG AGA TGT GAT ATT AGA A-3′ for CRMP-4 and 5′-*TAC TTC CAA TCC* ATG ACG AGT GAC CGT CTC CTT ATC-3′ and 5′-*TAT CCA CCT TTA CTG TTA* GTC TGC CAT CTT CCT CCG TG-3′ for CRMP-4 ΔC (overhangs in italics). The full-length and ΔC versions of CRMP-4 were cloned and expressed in a similar manner. The primers contained an approximately 20 bp overhang corresponding to the recombination site in the expression vector. Ligation-independent cloning (LIC) was used to clone the PCR products into the pNIC28-Bsa4 vector (Gräslund *et al.*, 2008 ▶). The coding region is thereby extended by 23 additional residues at the N-terminus. This includes a hexahistidine tag (His tag) as well as a TEV protease cleavage site preceding the protein sequence. The resulting plasmids were transformed into *Escherichia coli* BL21(DE3)pLysS competent cells. ZYP-5052 auto-induction rich medium (Studier, 2005 ▶) supplemented with the corresponding antibiotics kanamycin (100 µg ml^−1^) and chloramphenicol (34 µg ml^−1^) was inoculated with a pre-culture of these cells for expression. The cells expressing CRMP-4 and CRMP-4 ΔC were kept under constant agitation at 21°C for 24 h of growth. For full-length CRMP-4, the cells were kept at 37°C during the first 2 h of growth. The cells were then harvested by centrifugation at 4°C and the resulting pellets were either used immediately or frozen and stored at −20°C.

### 2.2. Protein purification   

For lysis, cell pellets were resuspended in ice-cold buffer *A* [50 m*M* HEPES pH 7.5 (25°C), 300 m*M* NaCl, 10 m*M* imidazole, 1 m*M* DTT, 10%(*v*/*v*) glycerol] supplemented with 1 mg ml^−1^ lysozyme, 5 µg ml^−1^ DNase and one cOmplete EDTA-free protease-inhibitor cocktail tablet (Roche) per 30 ml of resuspended cells and incubated for 40 min at 4°C. Cells were lysed further using sonication. The lysate was cleared by centrifugation at 38 000*g* for 30 min at 4°C. Then, 1 ml of Ni–NTA agarose beads (Qiagen) per 10 g of cell pellet was added to the supernatant. After incubation under constant agitation at 4°C for 40 min, the Ni–NTA agarose beads were collected by filtration and washed with 20 ml buffer *A*. Elution of the CRMP-4 and CRMP-4 ΔC proteins was achieved by a stepwise increase in imidazole concentration from 50 to 1000 m*M* in buffer *A*. The main fractions containing CRMP-4 ΔC were pooled together. TEV protease was added in a 1:100 molar ratio to remove the His tag, and the solution was dialyzed overnight against cleavage buffer [20 m*M* Tris pH 8.0 (25°C), 150 m*M* NaCl, 5 m*M* DTT] at 4°C. For the purification of full-length CRMP-4, the cleavage of the N-terminal tag and subsequent dialysis was omitted owing to the presence of the flexible protease-susceptible C-terminal domain. Both CRMP-4 protein variants were concentrated and, for further purification, applied onto a gel-filtration column (Superdex 200, GE Healthcare) which was pre-equilibrated with buffer *B* [50 m*M* HEPES pH 7.5 (25°C), 300 m*M* NaCl, 10%(*v*/*v*) glycerol]. The fractions containing the major peaks of CRMP-4 and CRMP-4 ΔC were pooled and concentrated to approximately 15 and 18 mg ml^−1^, respectively. The proteins were either used for crystallization immediately or flash-frozen in liquid nitrogen and stored at −80°C until further use.

### 2.3. Crystallization, structure determination and refinement   

Crystallization screening of both the CRMP-4 and CRMP-4 ΔC proteins was performed using the sitting-drop vapour-diffusion method. Sparse-matrix screens were set up utilizing a Phoenix robot (Dunn Labortechnik). Initial hits were optimized by varying the concentration of the precipitant and the pH of the buffer. Two crystal forms were obtained for CRMP-4 (*A* and *B*). Form *A* crystals of CRMP-4 grew fully within one week in 15%(*w*/*v*) PEG 6000, 100 m*M* Na HEPES pH 7.5, 100 m*M* KCl. Crystal form *B* grew within two weeks in 23%(*w*/*v*) PEG 3350, 200 m*M* sodium tartrate, 100 m*M* bis-tris propane pH 7.0, 50 m*M* sodium fluoride. For both crystallization conditions protein at 15 mg ml^−1^ concentration was mixed in an equal ratio with reservoir solution. Similarly, CRMP-4 ΔC crystallized within a week in 30%(*w*/*v*) PEG 3350, 100 m*M* sodium tartrate, 100 m*M* bis-tris propane pH 6.5 using a protein concentration of 18 mg ml^−1^. CRMP-4 crystals were usually cryoprotected by soaking the crystals in reservoir solution supplemented with 25%(*w*/*v*) ethylene glycol, but in some cases no cryoprotectant was used. For the CRMP-4 ΔC crystals no additional cryoprotection solution was needed. X-ray diffraction data were collected at 100 K at the I911-3 station at MAX-lab, Lund, Sweden (all crystal forms) and on beamline ID23-1 at ESRF, France (CRMP-4 form *B*). The data sets were processed with *MOSFLM* (Leslie & Powell, 2007 ▶) and scaled using *AIMLESS* (Evans, 2011 ▶) (Table 1 ▶). The structure of CRMP-4 ΔC was solved by molecular replacement using *Phaser* (McCoy *et al.*, 2007 ▶). A monomer of CRMP-5 (PDB entry 4b91; Ponnusamy & Lohkamp, 2013 ▶) was used as the search model after the sequence of the structure was adjusted to more closely resemble CRMP-4 using *CHAINSAW* (Stein, 2008 ▶). The initial molecular-replacement solution had an *R* value of 34.9%. Model building was performed using *WinCoot* (Emsley *et al.*, 2010 ▶). The structure of CRMP-4 in both crystal forms was solved by molecular replacement using *Phaser* with the CRMP-4 ΔC structure as a model. Reciprocal-space refinement was performed using *REFMAC*5 (Murshudov *et al.*, 2011 ▶). Twin refinement was used for the structure of crystal form *A*. The structure of crystal form *B* was refined using intensities corrected for lattice-translocation disorder (details are given below). The atomic coordinates and structure factors of CRMP-4 forms *A* and *B* and CRMP-4 ΔC have been deposited in the Protein Data Bank with accession codes 4cnt, 4cnu and 4cns, respectively.

### 2.4. Homology modelling and figures   

A homology model for CRMP-3 was built using the *SWISS-MODEL* web server (Arnold *et al.*, 2006 ▶) and the structure of the CRMP-2 tetramer (PDB entry 2gse; Stenmark *et al.*, 2007 ▶) as a template.

Figures were prepared using *CCP*4*MG* (McNicholas *et al.*, 2011 ▶) and *PyMOL* (v.1.5.0.4; Schrödinger).

## 3. Results and discussion   

### 3.1. Detection of the lattice-translocation disorder   

**Figure 1:**
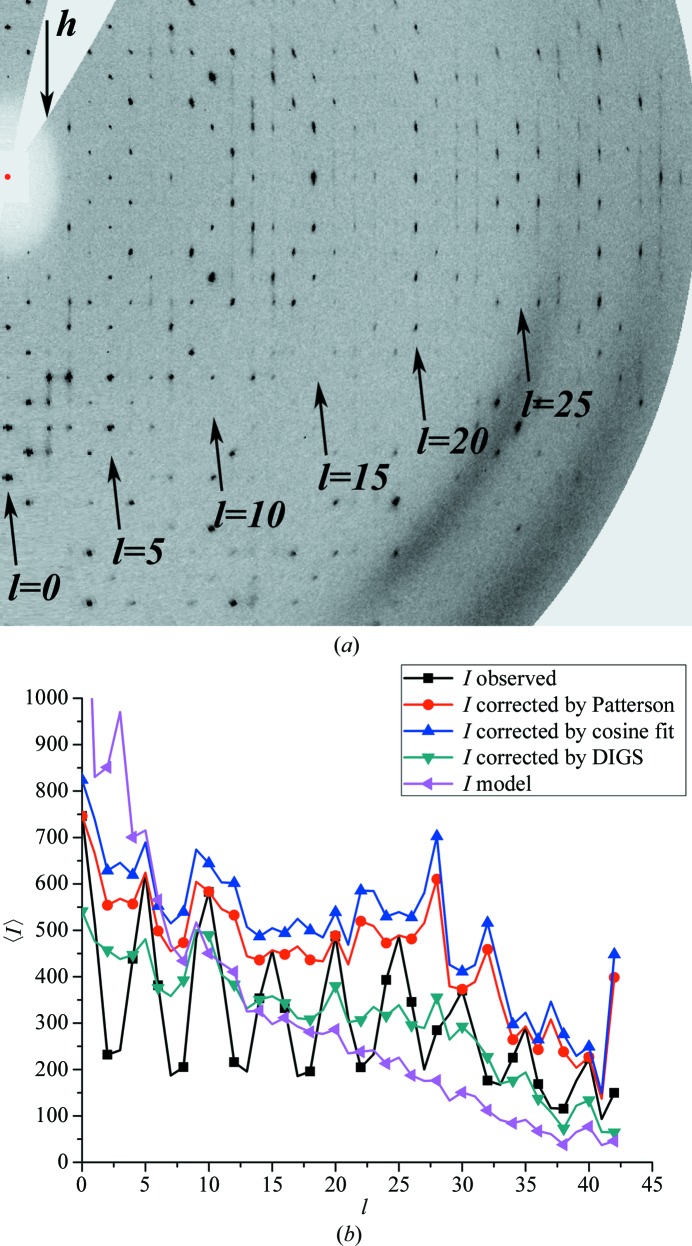
(*a*) Typical pseudo-precession diffraction pattern from CRMP-4 crystals (form *B*). The *h*0*l* plane is shown. Sharp reflections are observed along *h* for *l* = 5*n*. The other reflections are diffuse, with the streaks being most prominent at *l* = 5*n* + 2 and *l* = 5*n* + 3. *LABELIT* (Sauter *et al.*, 2004 ▶) was used to calculate the diffraction image from the experimental data and to produce the figure. (*b*) Layer-averaged intensities before and after different corrections. A similar curve for calculated intensities is shown for comparison. Observed intensities show clear modulation, with every 5*n* layer being strong (corresponding to the *l* layers with sharp Bragg spots). The different corrections remove the modulation and improve the fit to the calculated data, with DIGS being more efficient.

**Figure 2:**
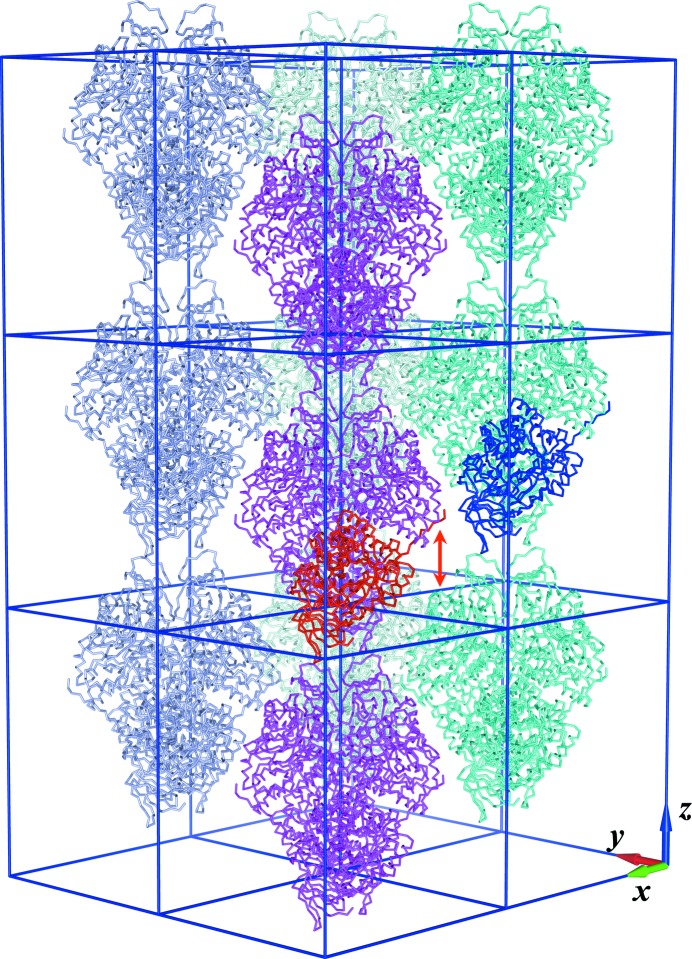
The crystal packing, tNCS and lattice-translation disorder in crystal form *B*. The two monomers related by tNCS, which form the asymmetric unit of an ordered *P*2_1_2_1_2 crystal domain, are shown in red and blue. The tNCS vector corresponds to the largest non-origin Patterson peak. There are two distinct packing possibilities for two adjacent vertical rows of tetramers. The tops of the tetramers from one row can pack against the bottoms of the tetramers from another row, as shown for the magenta and cyan rows, respectively. Alternatively, the tetramers from these rows could pack bottoms against tops, with the magenta row shifted down along *z* by 24 Å. The shift vector **t**_d_ is shown by the red arrow and corresponds to the second largest non-origin Patterson peak. In the first approximation, the contacts between rows are geometrically identical in the two packing modes although made by different pairs of tetramers. This packing ambiguity is responsible for translocation defects with translocation vector **t**_d_.

The diffraction patterns of all CRMP-4 crystals of form *B* showed diffuse reflections independent of the cryoprotection used. The spots at higher resolution were particularly smeared. However, some spots at lower resolution appeared to be sharp Bragg reflections, indicating that these diffuse features cannot be solely attributed to thermal disorder. Pseudo-precession diffraction images, simulated from the experimental data, showed a pattern of alternating layers of sharp Bragg spots and streaky reflections (Fig. 1 ▶ *a*). As a rule, sharp spots were observed for *l* = 5*n* and streaky diffraction spots for layers in between. The spots for *l* = 5*n* + 2 (and *l* = 5*n* + 3) were particularly streaky. However, for some data sets the sharpness of the Bragg spots at *l* = 5*n* diminished at higher resolution. This observation is in agreement with the plot of the mean intensity in the *l* layer *versus* *l* (Fig. 1 ▶ *b*), where intensities for *l* = 5 are particularly strong and those for *l* = 5*n*+ 2 (and *l* = 5*n* + 3) are weak. Furthermore, all data sets for these crystals showed anisotropic diffraction, which in some cases was very severe. The diffraction was stronger in the *l* direction and weaker in the *k* and particularly the *h* directions, which again is at least partially related to the observed spot characteristics. Analysis of the native Patterson function for all data sets collected for crystal form *B* revealed two strong non-origin peaks. The peak heights for these were between 60 and 71% and between 24 and 33%, respectively (Table 2 ▶). The fractional coordinates of the higher peak are approximately (0.5, 0.5, 0.4) and those for the second, lower peak are approximately (0, 0, 0.2) (Table 2 ▶). Cell-content analysis confidently predicted two molecules in the asymmetric unit with a Matthews coefficient of 2.48 Å^3^ Da^−1^ and a solvent content of 50.4%. This indicated that one of the peaks might be attributed to tNCS. The second peak corresponds to a shift of approximately 24 Å along *z* (Fig. 2 ▶). Since CRMPs are 50–70 Å in diameter, this shift would lead to a physically impossible overlap. Hence, this peak must originate from some other interference term caused by translationally related scatterers. In addition, the tNCS alone could not explain the pathological height of the strongest non-origin peaks. Based on the same size-of-molecule reasoning, and as opposed to the case of nearly exact pseudo-centring with a Patterson peak at (0.5, 0.5, 0.5), only different pairs of molecules can contribute to the equivalent peaks at (0.5, 0.5, 0.4) and (0.5, 0.5, 0.6). Therefore, the heights of the latter peaks cannot exceed half of the height of the origin peak and amount to 0.6. In principle, Patterson peaks would have been observed at the same positions if the crystal was twinned by reticular merohedry with twin index 5. However, this possibility was ruled out as there were no unaccounted spots during integration of images. The possibility of a different space group was also excluded since data scaled in lower symmetry showed the same features.

**Figure 3:**
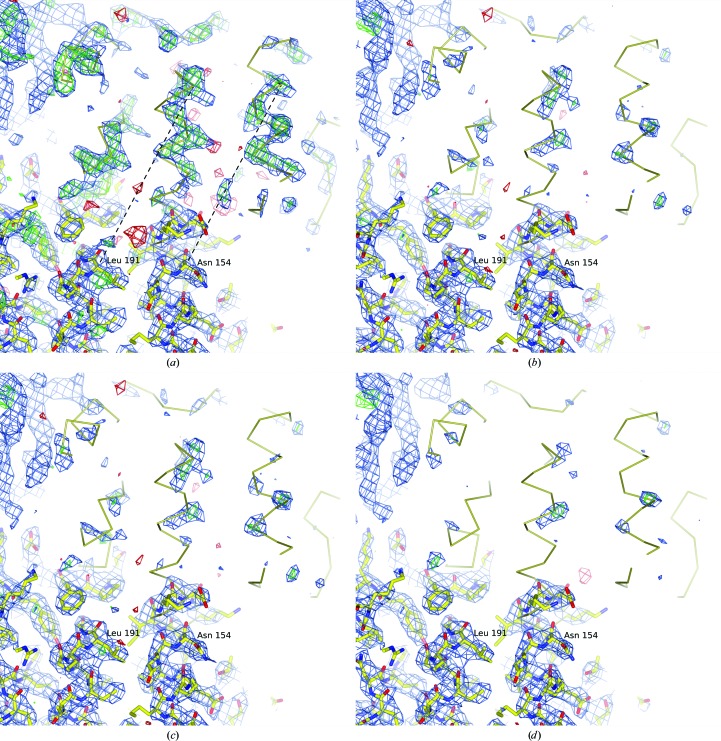
Model and electron-density maps (blue, 2*F*_o_ − *F*_c_, 0.25 e^−^ Å^−3^, 1.15 r.m.s.d.; green and red, *F*_o_ − *F*_c_, ±0.16 e^−^ Å^−3^, 2.8 r.m.s.d.) using no or different corrections of lattice-translocation disorder. The final model is shown in atomic stick representation. A C^α^ trace shows this model translated by **t**_d_, the translocation vector in (1), which also represents the vector between origin and non-origin Patterson peaks. Dotted lines connecting two helices indicates this translation vector. The second trace is shown to illustrate that the ghost density is a translated copy of the actual density. (*a*) Map originating from refinement without using any correction. Clear ghost density is visible. (*b*) Maps from simple correction, *i.e.* demodulation using the cosine function. (*c*) Maps resulting from correction using the Patterson map flattening protocol. (*d*) Maps resulting from DIGS (§[Sec sec3.3]3.3).

**Figure 4:**
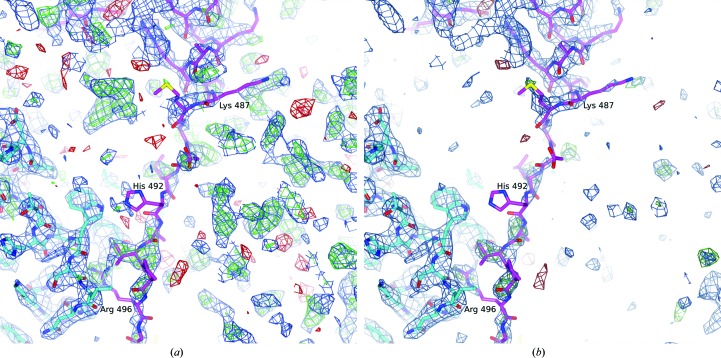
Correction of intensities reveals new traces. The final model of CRMP-4 (crystal form *B*) and OMIT electron-density maps are shown. The C-­terminal residues from 489 onwards of chain *B* (magenta C atoms) were excluded from the map calculations. (*a*) The electron density calculated using nonmodified data. Unambiguous tracing of the C-terminal residues is not possible owing to spurious ghost density. (*b*) Electron density from DIGS-corrected data. The C-terminal residues can be traced. The 2*F*_o _ − *F*_c_ maps contoured at 0.24 e^−^ Å^−3^ (1.1 r.m.s.d.) and *F*_o _ − *F*_c_ maps contoured at ±0.16 e^−^ Å^−3^ (±2.8 r.m.s.d.) are shown in blue and green/red, respectively.

More than five data sets were collected from CRMP-4 crystals of form *B* and all showed the same diffraction pathology and non-origin peaks in the Patterson maps. However, structure solution by molecular replacement was possible using these pathological data and solutions for two monomers in the asymmetric unit related by tNCS were found. The search model included only the core residues of CRMP, excluding residues from the C-terminal domain. The resulting electron-density map showed extensive unmodelled protein-like features (Fig. 3 ▶ *a*), which may be attributed to the missing residues in the search model. Automated model building with *Buccaneer* (Cowtan, 2006 ▶) was successful in building approximately 200 residues into this density. However, closer inspection of the newly built chain showed that it had essentially the same structure as the adjacent half of the closest monomer. This monomer and the partial model were related by a fractional translation of 0.2 along *z*, which is in accordance with the Patterson peak at (0, 0, 0.2) (Fig. 2 ▶). Several residues of the initially omitted C-terminal domain were visible and could also be built (Fig. 4 ▶ *a*), but its further extension was hampered by the apparent density from the non-existent molecule. Such ghost density strongly suggests the presence of the interference term in the observed intensities arising from lattice-translocation disorder.

Combining the experimental information from the sharp/streaky diffraction pattern, unexplained native Patterson peaks and the ghost density from shifted molecules strongly indicates that the data exhibit severe lattice-translocation disorder. The presence of a Patterson peak at (0, 0, 0.2) implies that the equivalent molecules at the opposite sides of a translocation defect are shifted relative to each other by about one fifth of the *c* cell dimension and explains the sharp Bragg spots at *l* = 5*n*. To obtain a cleaner electron-density map, which could potentially reveal residues from the as yet unresolved C-terminal domain, correction of the intensities by demodulation was undertaken.

### 3.2. Correction of intensities using lattice-translocation theory   

The packing of crystal form *B* is shown in Fig. 2 ▶ and, in addition, a possible alternative position of a row of tetramers is indicated. Geometrically identical interfaces for the two packing modes might suggest their nearly equal probabilities. A difference between the two probabilities arises from small conformational changes defined by the positions of tetramers in the second coordination sphere. The mean size of the ordered domains decreases with the decrease of this difference. In turn, when the domain size decreases and becomes comparable with the coherence radius of X-rays, the interference contribution to the scattered intensity from adjacent domains is no longer negligible. This results in streaky reflections and modulation of intensities, as in the case of the CRMP-4 form *B* crystals in this study.

In principle, it should be possible to provide an idealized stochastic model of the X-ray diffraction by such a partially disordered crystal. However, it is difficult to design a model that would carefully account for all experimental parameters, including data-collection and data-processing geometries (*e.g.* the orientation and dimensions of the integration box). Therefore, it is common practice to use standard data processing followed by correction of the data using a simplified model of the partial disorder. For example, Wang, Kamtekar *et al.* (2005 ▶) used a two-lattice treatment of the data affected by translocation defects. In this model, two adjacent domains are expanded to restore the global translational symmetry. Such a synthetic crystal contains two copies of the same structure related by the translocation vector **t**_d_ and with different occupancies, also denoted as the lattice-defect fractions κ and (1 − κ). In this model, the total intensity *I*_total_ of a given Bragg reflection **h** can be calculated from the intensity scattered by a single crystal, *I*_unit_, as follows:
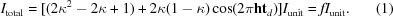
In (1) two parameters remain unknown: the translocation vector **t**_d_ and the fraction κ. The vector **t**_d_ can be determined with reasonable accuracy from the non-origin Patterson peaks. The fraction κ can be determined by a least-squares fit of (1) to the observed data. Under special conditions it can also be determined from the peak-height ratio (Wang, Kamtekar *et al.*, 2005 ▶). In other approaches, κ may be determined by trial and error from the subsequent structure-determination process or systematically until the Patterson map is free of significant non-origin peaks (Hare *et al.*, 2009 ▶; Zhu *et al.*, 2008 ▶). Once the parameters are known (1) can be used to correct the observed intensities.

Using the least-squares fitting method, we determined κ to be 0.205 for data set 1. The translocation vector **t**_d_ was obtained from the Patterson peak position (Table 2 ▶). A model illustrating the corresponding translocation disorder is given in Fig. 2 ▶. The resulting electron density (Fig. 3 ▶ *b*) and refinement statistics (Table 3 ▶) were substantially improved. Using the method in which κ is obtained *via* reduction of the non-origin Patterson peak, the fraction κ was determined to be 0.20 (variation of κ in steps of 0.01). As expected, the two methods gave similar results, and correction of the intensities using the second method similarly improved both the quality of the electron-density maps (Fig. 3 ▶ *c*) and the refinement statistics (Table 3 ▶). Naturally, this is also reflected in the layer-averaged intensity distributions, which are void of the modulation after correction (Fig. 1 ▶ *b*).

### 3.3. Further correction of intensities using the modelled crystal structure   

The procedure described in the previous section removed modulations with a period of approximately 5 along *l* (Fig. 1 ▶ *b*). However, substantial differences remained between the observed demodulated intensities averaged over *h* and *k* and the similar curve calculated from the atomic model. Therefore, we undertook an attempt to improve the demodulation procedure, and in particular to use the intensities from the refined model as a reference for fitting modulation parameters.

**Figure 5:**
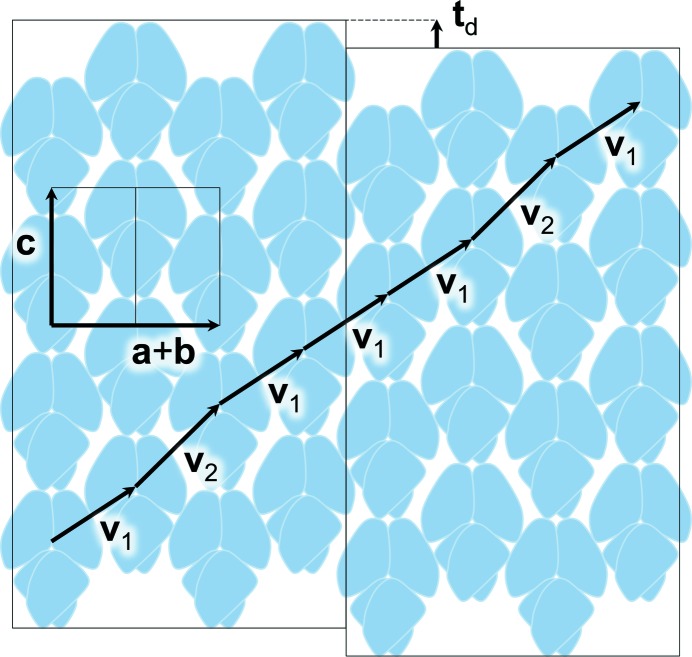
A model of the lattice-translocation disorder. Tetramers are shown as blue shapes with white lines indicating the approximate contours of monomers. As in Fig. 2 ▶, two adjacent columns of tetramers can be related by one of the two alternative translation vectors, **v**_1_ or **v**_2_. Thin black rectangles indicate ordered domains with local *P*2_1_2_1_2 symmetry, in which **v**_1_ and **v**_2_ alternate. Sequences of repeated vectors (here, **v**_1_ **v**_1_ **v**_1_) occur at lattice-translocation defects. The translocation vector **t**_d_ defines the relative shift of neighbouring domains from the position in which they would have formed a continuous single crystal.

Fig. 5 ▶ presents a scheme of the putative structure of a defect area of the partially disordered crystal. The translocation defect with the translocation vector **t**_d_ is responsible for the lack of global translation in the direction **a** + **b**. Symmetry considerations suggest that an identical scheme can be drawn for the section defined by vectors **c** and **a** − **b**, implying a two-dimensional disorder. Lack of global translation in the directions **a** + **b** and **a** − **b** is supported by the presence of halos around the Bragg reflections in the views along c* (Supplementary Fig. 1^1^). These halos are seen as diffuse streaks in the views orthogonal to c* (Fig. 1 ▶ *a*). In addition, such two-dimensional disorder is in agreement with the parameters of anisotropic diffraction, because the drop of the mean intensity with resolution is faster along a* and b* than along c*.

For simplicity, we consider a partially disordered structure in which the global crystallographic translation is only missing in the direction **a** + **b**. Generalization to the actual two-dimensional disorder is straightforward but it would not change the results of this section. Three structures are considered to describe the modulation of the intensities and the demodulation procedure. The first structure (further referred to as structure *L*) is a structure of a single layer represented by one column of tetramers in Fig. 5 ▶. We assume that this structure has an exact two-dimensional translation symmetry with base vectors **c** and **a** − **b**. The second (*C*) is a refined *P*2_1_2_1_2 model structure which corresponds to an ordered domain of the disordered structure. The third (*O*) is a partially disordered structure containing the defects shown in Fig. 5 ▶. This structure corresponds to a real partially disordered crystal. The structures *C* and *O* are considered to be composed from identical layers *L*; in this approximation, any small conformational differences between molecules with different contact patterns are disregarded. Different X-ray scattering patterns correspond to the structures *L*, *C* and *O* (lines, spots and streaky spots, respectively), but we are interested only in intensities at the points where crystal structure *C* has its Bragg spots. We denote the three sets of intensities at the Bragg positions as *I_L_*, *I_C_* and *I_O_*, respectively.

As follows from the schematic in Fig. 5 ▶, the translocation vector **t**_d_ is collinear with **c**,
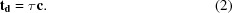
Here τ ≃ 0.2, and the vectors **v**_1_ and **v**_2_ are expressed in terms of **t**_**d**_ and the base crystallographic translations as follows:
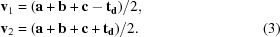
Two arbitrary layers *L* and *L*′ are related by a linear combination of **v**_1_ and **v**_2_ with integer coefficients *j*_1_ and *j*_2_, 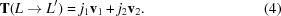
From (2–4) it follows that
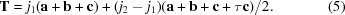
Hence,
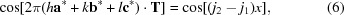
where
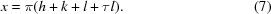
Accordingly, *I_O_* is expressed in terms of *I_L_* as follows,
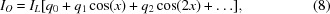
where *x*, *I_L_* and *I_O_* depend on *hkl*, while the coefficients *q_n_* are proportional to the probability of finding |*j*_2_ − *j*_1_| = *n* for two randomly selected layers. The separation between these layers has to be less than the coherence radius of the X-rays.

In the ordered *P*2_1_2_1_2 domains (Fig. 5 ▶), as well as in the structure *C*, the vectors **v**_1_ and **v**_2_ alternate. Therefore, |*j*_2_ − *j*_1_| equals 0 or 1 with equal frequency and
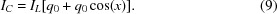
Coefficients between *I_O_* and *I_C_* are nonlinear functions of cos(*x*), 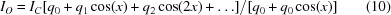
and cannot be made linear in a general case. This is a likely reason for the large discrepancy between the demodulated intensities and intensities from the *P*2_1_2_1_2 model if the demodulation procedure is performed according to (1). In addition, (8) describes the diffraction from the whole multi-domain crystal, as opposed to, for example, the two-domain (two-lattice) approximation used in (1).

The equations in this section relate the intensities at Bragg points, and any combinatorial estimate for coefficients *q* would work reasonably well only if the integration box for spot integration is small. In practice, the size of the box will affect the relative values of *q* as well as the overall anisotropic *B* factor. The coherence radius of an X-ray source is another factor that must be accounted for in any combinatorial estimate of these coefficients. Of course in practice accurate theoretical values of *q* are not needed, as they can simply be considered as unknown parameters (together with τ) in a nonlinear statistical model defined by (10). It is also possible to use the structure *L* as a reference and (8) for fitting of *I_O_* and *I_C_* (*L* is a substructure of *C* and is available as soon as *C* is available), thus avoiding small values that may occur in the denominator of (10). However, any fitting, linear or nonlinear, where the response variables are the intensities requires accurate weighting of residuals, otherwise the fitted values will mainly be defined by a few strong intensities. This problem is well known in crystallographic refinement and, for example, an accurate weighting of residuals is critical for least-squares minimization against intensities implemented in *SHELXL* (Sheldrick, 2008 ▶).

Therefore, we used an even more simplified algorithm, DIGS. Here, the observations were grouped by two factors, the value of *l* and the parity of *p* = *h* + *k*. As follows from (7) and (10), the coefficients relating *F_O_* to *F_C_* (as well as the coefficient relating *I_O_* to *I_C_*) should be the same for all reflections from the same group. These can be used as unknown parameters in linear fitting, provided that each group contains a significant number of observations. There were 80 (*i.e.* 2*l*_max_) such groups in data set 1, with an average of 319 reflections per group and only eight groups with less than 100 reflections. The estimates of these 80 parameters were used for demodulation. The entire refinement protocol using DIGS included the following two alternating steps.

(i) Refinement of the current model *C* ^(*n*−1)^ of the structure *C*, where *n* is the iteration number, against the current demodulated set of structure amplitudes *F_O_* ^(*n*−1)^ using *REFMAC*5 to generate a new model *C* ^(*n*)^ and a corresponding set of calculated structure amplitudes *F_C_* ^(*n*)^.
(ii) For each group with the same overall modulation coefficient (with the same parity of *p* and value of *l*), scaling of the observed structure amplitudes *F_O_* to the calculated structure amplitudes *F_C_* ^(*n*)^ to generate a new set of demodulated structure amplitudes *F_O_* ^(*n*)^:
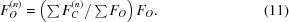

The procedure was repeated each time the model was corrected. Convergence was reached at *n* between 2 and 5, dependent on the stage of model rebuilding. Eventually, an *R* and *R*_free_ of 25.5 and 29.5%, respectively, between the final model and the final demodulated data were obtained. The iterative demodulation, refinement and model correction resulted in clearer electron density, which in turn allowed further model building and increased the correlation between the model and the experimental data (Table 3 ▶ and Fig. 4 ▶).

With this approach, any nonlinear fitting and any fitting involving intensities as predictor or response variables was avoided. We can envisage, however, that a more thorough approach, in which modulation equations are incorporated into the likelihood and weighting coefficient machinery of, for example, *REFMAC*5, would produce even better results and would not require simplifications as used in the discussed procedure.

### 3.4. CRMP-4 structures from different crystal forms   

#### 3.4.1. Quality of the structural models   

Two different version of CRMP-4, full-length (1–570) and ΔC (13–490), were heterologously produced in good yield in *E. coli*. TEV protease removed the N-terminal histidine tag of the CRMP-4 ΔC efficiently. To speed up the purification and thus avoid exposure to proteases, the N-terminal tag was deliberately not cleaved off for CRMP-4 since it comprises the flexible C-terminal region which appears to be proteolytically susceptible. However, full-length CRMP-4 could be separated efficiently from the proteolytically cleaved version by size-exclusion chromatography and this was used for crystallization (Supplementary Fig. S2). CRMP-4 was crystallized in two different crystal forms, form *A* belonging to space group *P*2_1_ and form *B* belonging to space group *P*2_1_2_1_2. CRMP-4 ΔC crystallized in space group *P*2_1_. All structures were determined by molecular replacement. Firstly, CRMP-4 ΔC was solved using a modified model of CRMP-5 ΔC as a search model. The refined model of CRMP-4 ΔC was then used for molecular replacement of the full-length versions.

The crystal of CRMP-4 ΔC contains a tetramer in the asymmetric unit. Almost all of the residues are resolved in the electron density. Residue 490 is missing in all chains and residue 489 is missing in chains *A* and *D*. In chains *C* and *D* the serine from the N-terminal TEV cleavage site is also visible. The model was refined to an *R* factor of 19.1% and a free *R* factor of 22.6% (Table 1 ▶) and has excellent quality as indicated by *MolProbity* (100th percentile; Chen *et al.*, 2010 ▶). One residue, Gly357, is found just outside the Ramachandran plot, as has been observed for other CRMPs (Ponnusamy & Lohkamp, 2013 ▶). CRMP-4 (form *A*) was solved from a twinned crystal. The data scaled in the orthorhombic space group *C*222; however, the model could not be refined. Careful analysis of intensity statistics showed that the crystal belonged to space group *P*2_1_ and was twinned. The twin fraction was refined to 31%. With structure factor-based twin refinement, the final *R* and *R*_free_ converged to 18.7 and 23.6%, respectively (Table 1 ▶). The model consists of a tetramer in the asymmetric unit. Residues 12–496 (or 497 in chain *B*) are resolved in the electron density. The electron density for the 6–8 C-terminal residues is weak (from residue 490 in chains *A* and *C* and residue 489 in chains *B* and *D*) but is sufficiently well defined to build a good model. The model of CRMP-4 from crystal form *A* has very good quality as indicated by *MolProbity* (Table 1 ▶).

CRMP-4 from crystal form *B* was initially solved with uncorrected data. However, owing to the lattice-translocation disorder the refinement was performed against DIGS-corrected data as described above. The model consists of a dimer with the two monomers in the asymmetric unit related by tNCS. The crystallographic twofold symmetry relates two dimers which form the biologically relevant tetramer (Fig. 2 ▶). Both monomers in the model consist of residues 12–499. CRMP-4 from crystal form *B* has good quality as calculated by *MolProbity* (Table 1 ▶).

Crystals of CRMP-4 contain primarily the full-length protein as shown by SDS–PAGE of dissolved crystals (Supplementary Fig. S3). Cell-content analysis for crystal form *A* (values in parentheses are for form *B*) gives a Matthews coefficient of 2.18 (2.48) Å^3^ Da^−1^ and a solvent content of 43.6% (50.4%) assuming that the full-length protein was crystallized, whereas values of 2.58 (2.94) Å^3^ Da^−1^ and 52.4% (58.1%) are calculated assuming that the crystal contains CRMP-4 ΔC. Crystal packing in both crystal forms show that the truncated C-terminus points towards the solvent region, where sufficient space is available for the disordered residues (Fig. 2 ▶). Hence, it seems very likely that although full-length protein is present in the crystal the C-terminal tail is dis­ordered and cannot be resolved in the electron density.

#### 3.4.2. Overall structure of CRMP-4   

**Figure 6:**
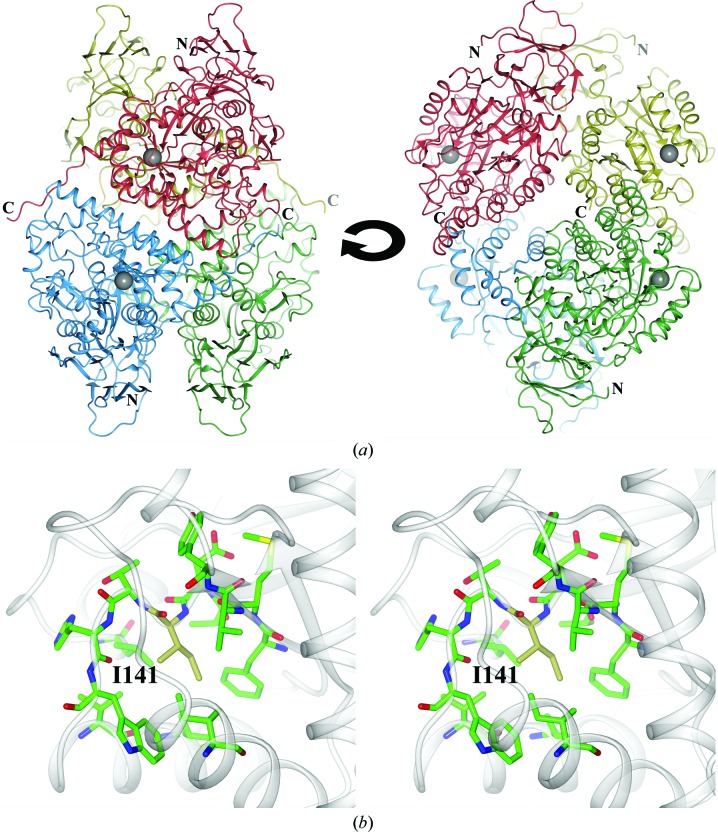
Location of the I141V mutation of CRMP-4 in ALS patients. (*a*) Ribbon presentation of the CRMP-4 tetramer. The arm–lever interface is formed by, for example, chains *A* and *B* (red and blue) and the arm–arm interface by, for example, chains *A* and *D* (red and gold). The position of the I141V mutation is indicated by grey spheres. (*b*) Close-up stereoview of the I141V mutation. The mutated Ile is shown in yellow stick representation, with the surrounding interacting residues in green. Mutation of Ile to Val would result in a small void in the shown hydrophobic cluster.

Based on sequence similarity of the core region (residues 64–413), CRMP-4 and other members of the CRMP family are assigned as metal-dependent hydrolases in the Amidohydro_1 subfamily within the amidohydrolase superfamily (Pfam family PF01979; Punta *et al.*, 2012 ▶). The amidohydrolase domain of CRMP-4 displays a typical triosephosphate isomerase-like barrel fold. It is capped by a smaller β-sandwich domain composed of residues from both the N-terminus and the C-terminus (13–63 and 414–455; Fig. 6 ▶ *a*). This domain has no significant structural similarities outside the amidohydrolase family and no functional homologue in the Sema3A signalling pathway (Deo *et al.*, 2004 ▶). The structure of CRMP-4 closely resembles the enzyme DHPase, which also belongs to the amidohydrolase superfamily. However, to date no DHPase activity has been observed for any of the CRMP family members (Ponnusamy & Lohkamp, 2013 ▶; Wang & Strittmatter, 1997 ▶). As observed previously in CRMP-5, the majority of the C-terminal residues of CRMP-4 (497/500–570) were not visible in the crystal structure. Only 11 more residues, up to residue 499, were revealed with the CRMP-4 structure, which form an extended loop protruding from one protomer towards the other (Figs. 2 ▶ and 6 ▶). The extended loop is anchored by a conserved salt bridge between the guanidinium group of Arg496 and the carboxyl group of Glu221 residing in α-helix 6 (Fig. 4 ▶). Similar C-terminus anchoring salt bridges were observed in full-length CRMP-5 and bacterial DHPase from *Sino­rhizobium meliloti* (Martínez-Rodríguez *et al.*, 2010 ▶; Ponnusamy & Lohkamp, 2013 ▶). The location and conformation of the three most distal C-terminal residues, Gly497, Met498 and Tyr499, were resolved for the first time in CRMP-4, but only in crystal form *B*. These residues extend the C-terminal loop further in the same direction and pack well against the hydrophobic Trp295 of the neighbouring monomer.

The asymmetric units of CRMP-4 form *A* and CRMP-4 ΔC each contain four monomers forming a homotetramer with internal 222 symmetry. CRMP-4 form *B* contains two monomers forming a tetramer by crystallo­graphic twofold symmetry. All individual monomers are very similar to one another, with r.m.s. deviations between monomers from different crystal forms of around 0.1–0.4 Å (based on equivalent C^α^ positions). Similar homotetramers were observed for other CRMPs and for the enzyme DHPase. The homotetramer is formed by using two different dimer interfaces: monomers *A* and *B* (or *C* and *D*) form the ‘arm–lever’ interface and monomers *A* and *D* (or *B* and *C*) create the second ‘arm–arm’ interface (Fig. 6 ▶ *a*; Ponnusamy & Lohkamp, 2013 ▶). The arm–lever interface buries an average of 993 Å^2^ of accessible surface area per monomer and includes 4–5 hydrogen bonds and 2–3 salt bridges. The arm–arm interface buries slightly more surface area (1090 Å^2^ per monomer) and includes as many as 14–16 hydrogen bonds and four salt bridges. However, the smaller number of noncovalent bonds is compensated in the arm–lever interface by burying more hydrophobic residues (solvation free energy of −11.1 kcal mol^−1^ compared with −2.3 kcal mol^−1^ for the arm–arm interface) as calculated using *PISA* (Krissinel & Henrick, 2007 ▶). The arm–lever interface is further strengthened in CRMP-4 by 11 additional noncovalent interactions and a buried solvent-accessible area of 2124 Å^2^ per monomer. This increase in interface interactions is mainly owing to the anchoring of the extended loop by Arg496 and the three additional C-terminal hydrophobic residues. Disruption of the salt bridge by mutation of the equivalent Arg in a bacterial DHPase did not have any effect on tetramer assembly (Martínez-Rodríguez *et al.*, 2010 ▶). However, this salt bridge may have a crucial role in positioning the flexible C-terminus present in CRMPs. The latter is predicted to be intrinsically unstructured and devoid of any defined secondary-structure elements in all CRMPs and is important in regulation and binding of several interaction partners (Yamashita & Goshima, 2012 ▶).

Whilst conducting this research, a crystal structure of human CRMP-4 was deposited by the Structural Genomics Consortium (PDB entry 4bkn) with no accompanying publication. The protein was produced in insect cells, in contrast to our study which used a bacterial expression system. As observed here and previously for other CRMPs, only residues 13–488 and 493–496 could be resolved and the C-terminal domain remains elusive. The space group for PDB entry 4bkn is *C*2, with two molecules in the asymmetric unit forming a dimer *via* the arm–arm interface. A tetramer is assembled by crystallographic symmetry. This arrangement and symmetry is different to the structures presented here. Nevertheless, the molecular structures are very similar to each other, with an r.m.s.d. of 0.23–0.30 Å between monomers and of 0.35–0.50 Å between tetramers.

#### 3.4.3. Homo- and hetero-oligomerization   

Members of the CRMP family prefer to form heterotetramers rather than homotetramers (Fukada *et al.*, 2000 ▶; Wang & Strittmatter, 1997 ▶). The molecular basis of the regulation and function of such hetero-oligomers remains elusive. CRMP-4 and CRMP-4 ΔC, similar to other CRMPs, form a homotetramer in the crystals using the arm–lever and arm–arm interfaces (see above). Using gel-filtration analysis, we identified CRMP-4 to exist as a tetramer in solution and CRMP-4 ΔC to exist as lower oligomers, possibly as monomers and dimers in equilibrium. The difference may be owing to concentration-dependent oligomerization as observed for CRMP-1, CRMP-2 and CRMP-5 (Ponnusamy & Lohkamp, 2013 ▶). The homo-tetramer of CRMP-4 is similar to the tetramers of CRMP-1, CRMP-2 and CRMP-5, with r.m.s. deviations of 0.6–1.5 Å (based on equivalent C^α^ atoms). Analysis and comparison of the homotetramer interfaces using *PISA* (Krissinel & Henrick, 2007 ▶) revealed that CRMP-1 is distinct from all other CRMPs. Both the arm–lever and arm–arm interfaces of CRMP-1 are different in regard to the number of buried hydrophobic residues and noncovalent bonds. The arm–arm interface is strongest in CRMP-2 and CRMP-5 followed by CRMP-4. In CRMP-1 this interface buries a small hydrophobic surface and despite having a comparable number of noncovalent interactions the interface is weak. Concomitantly the arm–lever interface in CRMP-1 is strong, including more hydrophobic and other noncovalent interactions. CRMP-2 and CRMP-5 have the weakest arm–lever interface, but incidentally a stronger arm–arm interface. In general, it appears that CRMP-2 and CRMP-5 prefer the arm–arm homo-interface and CRMP-1 prefers the arm–lever homo-interface. For CRMP-4 this is less clear as both interfaces appear similar. The overall high sequence conservation and similar tertiary structure between members of the CRMP family may allow them to form hetero-oligomers without the need for major conformational changes. However, with no structural details of hetero-oligomers available to date, it is difficult to precisely identify the residues that may contribute to the preferential formation of such hetero-oligomers, but it is worthwhile pointing out a few putatively significant residues based on the available homotetramer structures. Some of the residues determining such hetero-oligomer preferences have been described previously. For example, CRMP-1 is able to form hetero-oligomers with all other CRMPs except for CRMP-5 (Fukada *et al.*, 2000 ▶; Ponnusamy & Lohkamp, 2013 ▶; Wang & Strittmatter, 1997 ▶). Residues Asn237 and Lys265 make two hydrogen bonds with Glu223 in the arm–lever interface in CRMP-5. These residues are replaced by Gly and Pro, respectively, in CRMP-1 but not in other CRMPs, thus hampering the hydrogen bonding. Similarly, Lys473 in CRMP-5 makes three hydrogen bonds with residues Glu370, Asn371 and Thr23 in the arm–arm interface, and is replaced by Glu only in CRMP-1. Together with other small changes, the sequence alterations at these two crucial spots will weaken CRMP-1–CRMP-5 heterotetramer formation (Deo *et al.*, 2004 ▶; Ponnusamy & Lohkamp, 2013 ▶).

**Figure 7:**
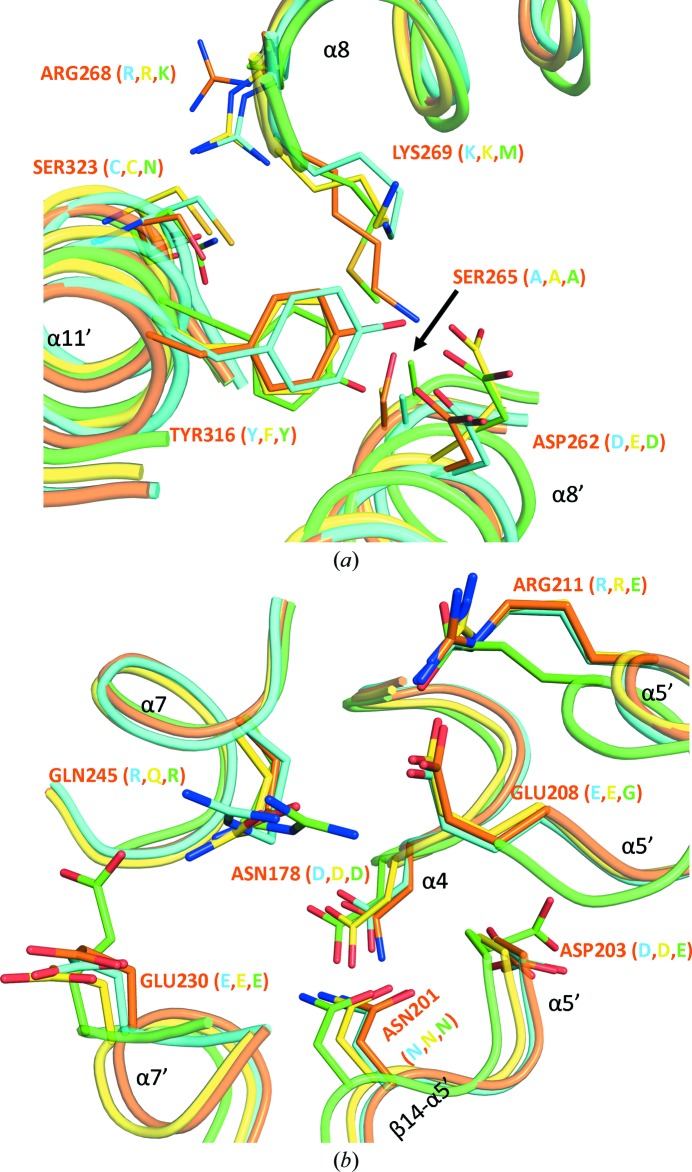
Residues that are potentially important in determining hetero-oligomerization and homo-oligomerization in CRMPs. The crystal structures of CRMP-1 (cyan), CRMP-2 (yellow) and CRMP-5 (green) are superimposed onto CRMP-4 (orange). Both interfaces, (*a*) arm–arm and (*b*) arm–lever, contain conserved and nonconserved residues. Secondary-structure elements and interacting residues are labelled for CRMP-4, with the corresponding residues of CRMP-1, CRMP-2 and CRMP-5 given in parentheses.

CRMP-4 was shown to form hetero-oligomers with all members of the family, making the identification of crucial preference-determining residues more challenging. In the arm–arm interface, there are two conserved positively charged residues at the end of α-helix 8, which are likely to be more involved in hetero-oligomerization rather than homo-oligomerization (Fig. 7 ▶ *a*). The first residue is Lys/Arg268, which is able to form a hydrogen bond to the carbonyl O atom of Ser323 present at the end of α-helix 11′ in CRMP-3 and CRMP-4 and an additional hydrogen bond to the side chain of the corresponding asparagine found in CRMP-5 (Supplementary Fig. S4). The second residue is Lys/Arg269, which is able to make up to three hydrogen bonds to Asp/Glu262 from α-helix 8′ and is absent only in CRMP-5. Another potential hydrogen-bonding partner Ser265 from α-helix 8′ is present in CRMP-4 but is replaced by Ala in other CRMPs. Found in its vicinity are further residues able to form interface hydrogen bonds, such as Tyr316 in CRMP-4 and His316 in CRMP-3. This residue, belonging to α-helix 11′, is substituted by Phe in CRMP-2, which thus loses a favourable interaction. Moreover, residue Tyr477 (otherwise Phe, His or Thr) from α-helix 16 in CRMP-4 slightly increases the hydrophilic nature of the arm–arm interface and provides potential hydrogen bonding with Arg372 from α-helix 14′ of CRMP-5. Additionally, Lys480 from α-helix 16, which is conserved in all CRMPs except for CRMP-1, where it is replaced by a less favourable Gln, makes several interactions with acidic residues present in the loop between α-helices 13′ and 14′ as described previously (Deo *et al.*, 2004 ▶). The number of residues differing between CRMP-4 and other CRMPs is smaller in the arm–lever interface than in the arm–arm interface. Asn178 present in α-helix 4 of CRMP-4 would make more favourable interactions with Asn201 and Asp/Glu203 (α-helix 5′ and the vicinity thereof) than the equivalent Asp in other CRMPs (Fig. 7 ▶ *b*). Residue 245 in α-helix 7 is either a Gln (CRMP-2, CRMP-3 and CRMP-4) or an Arg (CRMP-1 and CRMP-5). Whereas Gln can donate and accept hydrogen bonds, Arg is usually only able to donate them to the conserved residues Glu208, Arg211 and Glu230 from α-helices 5′ and 7′. In CRMP-5 only, residues 208 and 211 are substituted by Gly and Glu, respectively, thus losing some favourable interactions.

These few changes seen at the interface of CRMP-4 give an indication of, but cannot clearly define, the preference for hetero-oligomer over homo-oligomer formation. The preference of a specific hetero-oligomer, for example CRMP-1–CRMP-2 over CRMP-1–CRMP-4, may not be solely based on the sequence conservation and structure, but most likely would be influenced by and be synergistic with the varying temporal and spatial expression patterns of CRMPs (Gu *et al.*, 2000 ▶; Veyrac *et al.*, 2005 ▶; Wang & Strittmatter, 1996 ▶).

#### 3.4.4. Putative CRMP active site   

Although CRMPs are closely related to the enzyme DHPase, essential residues mediating DHPase activity are missing in CRMPs, including CRMP-4. Only two of the six residues crucial for metal binding to DHPases are present in CRMP-4, and the carboxylated lysine bridging the two zinc ions is replaced by leucine (Leu165). However, the active-site-like cavity is still present in CRMP-4 and other CRMPs (Deo *et al.*, 2004 ▶; Ponnusamy & Lohkamp, 2013 ▶; Stenmark *et al.*, 2007 ▶). With a pocket volume of around 893 Å^3^, it is more solvent-exposed and larger than the pockets in CRMP-1 (540 Å^3^) and CRMP-2 (580 Å^3^) and is considerably larger than that in CRMP-5 (277 Å^3^). Recently, CRMP-3, but not CRMP-4, was shown to exhibit histone H4 deacetylase activity which leads to E2F1-dependent neuronal death (Hou *et al.*, 2013 ▶). Histone deacetylases (HDAC) are grouped into four classes and are either NAD^+^- or Zn^2+^-dependent enzymes (Bottomley *et al.*, 2008 ▶; Lombardi *et al.*, 2011 ▶). The active site of the metal-dependent deacetylases contains a zinc ion coordinated by the side chains of two conserved aspartate residues and one histidine residue. Furthermore, a tyrosine or histidine residue is involved in the stabilization of the transition state. Given the high sequence identity between CRMP-3 and CRMP-2 (77%), a reliable model could be obtained for CRMP-3 (see above). In the modelled CRMP-3 structure important DHPase active-site residues are absent and are replaced by the same residues as observed in CRMP-4. At 693 Å^3^, the pocket volume is slightly smaller than for CRMP-4. Since there is no classical NAD^+^-binding site found in CRMPs, available CRMP models were scanned for potential Zn-binding sites using the *FEATURE* metal-scanning web server (http://feature.stanford.edu/metals/; Ebert & Altman, 2008 ▶). Several potential Zn-binding sites were identified in the active-site-like pocket in the available experimental and modelled CRMP structures. However, only one site was unique to CRMP-3 and similar to HDAC active sites. This putative binding site for a zinc ion is found at the entrance of the pocket, comprising residues His227 and Glu200. His223 and Tyr170 found in the vicinity might also take part in mediating deacetylase activity (Supplementary Fig. S5). In CRMP-4 and other CRMPs His227 is replaced by Arg, therefore blocking the potential zinc ion-binding site. Other predicted sites were not unique or lacked at least one of the crucial residues for HDAC activity, *e.g.* Tyr170. Nonetheless, this requires further experimental investigation.

#### 3.4.5. CRMP-4 I141V mutation in ALS patients   

Recently, a mutation in the *DPYSL3* gene encoding CRMP-4, c.421A>G (I141V; Supplementary Fig. S4), has been described in some ALS patient groups (Blasco *et al.*, 2013 ▶). Experiments in cell cultures expressing the CRMP-4 mutant variant showed impairment of motor neuron survival as well as axonal outgrowth. The replacement of an Ile by a Val is a very conservative mutation, which would not necessarily have a drastic impact on the structural integrity of the protein. In the CRMP-4 crystal structure the mutation site is located close to the surface of the tetramer (Fig. 6 ▶ *a*). The residue is central to a hydrophobic cluster in the core of the protein (Fig. 6 ▶ *b*). Owing to the missing C^δ^ atom the observed mutation would cause a small void in this cluster. It is possible that this void destabilizes the protein slightly and/or causes some changes in the adjacent surface for protein binding, which could explain the biological effect. Ile141 is strictly conserved within the CRMP family in humans and amongst CRMP-4s between species. However, in human DHP the equivalent residue (135) is a Val, but here several other residues are altered in the cluster to compensate for the missing C^δ^. Further *in vivo* and *in vitro* studies using purified mutant protein will help to explain the observed functional impairment.

## 4. Conclusions   

The structure of human CRMP-4 is a valuable new addition to the library of human CRMP family structures, with CRMP-4 being the fourth of the five members to be structurally characterized. While it still does not provide a final explanation for the prevalence of hetero-oligomer over homo-oligomer formation by CRMPs, a number of important interactions at the tetramerization interfaces have been revealed. Furthermore, our results shed light on disease-associated mutations, which can now be investigated further, such as those observed in patients with the motor neuron disease ALS. Structural analysis helped to further account for the lack of DHPase-like enzymatic activity in CRMPs, in spite of the overall structural similarity between CRMPs and DHPases. At the same time, we suggest a possible explanation for the residual HDAC activity of CRMP-3 based on a homology model calculated in the present study. However, future structural studies of the CRMP family, especially the structures of hetero-oligomers, would be required in order to better understand the physiological functions of this protein family in health and disease.

Notably, the presented structures are of importance not only owing to their biological impact, but also because one of them presents an interesting and unusual case of a crystal disorder that first manifested itself (after structure solution) as the presence of ghost-like electron density in a solvent region. Twin refinement, which was used for another crystal form, is now an almost routine procedure. In contrast, attempts to deal with partially disordered crystals, such as that encountered here, are undertaken much less frequently. In this study, demodulation of diffraction data from form *B* CRMP-4 crystals, which were affected by lattice-translocation disorder, was performed using two different techniques. The so-called lattice-alignment method, which is also suitable for subsequent *ab initio* structure determination, employs the integrated intensities only, with demodulation parameters being derived from the positions of non-origin Patterson peaks and subsequent nonlinear model fitting. A better consistency between observed and calculated intensities was achieved by a novel method, DIGS, that uses experimental data as well as calculated intensities and that does not rely on any particular parameterization. DIGS as described here can be applied to similar cases of crystal abnormality.

In addition, crystal form *B* of CRMP-4 may present a useful test case for the development of finer models of diffraction from partially disordered macromolecular crystals. One possibility is to consider periodic structures, in which one repeat contains, for example, several molecular layers and provides a model of both ordered domains and defects. Average intensities from an ensemble of such structures can be interpolated to describe actual reflection profiles. The intensities can either be estimated analytically from, for example, Markov chain approximations or be obtained using simulations (Weber & Bürgi, 2002 ▶), although at present simulations would be more suitable for small molecules owing to the high computational cost.

## Supplementary Material

Supporting Information.. DOI: 10.1107/S1399004714006634/dz5326sup1.pdf

PDB reference: CRMP-4 ΔC, 4cns

PDB reference: CRMP-4, 4cnt

PDB reference: 4cnu

## Figures and Tables

**Table 1:** Data collection, processing, structure solution and refinement Values in parentheses are for the outer shell.

	CRMP-4 (1570)		
	Crystal form *A*	Crystal form *B*	CRMP-4 C (13490)
Data collection			
Diffraction source	I911-3	I911-3	I911-3
Wavelength ()	1.07068	1.07068	1.04088
Temperature (K)	100	100	100
Space group	*P*2_1_	*P*2_1_2_1_2	*P*2_1_
Unit-cell parameters			
*a* ()	86.4	96.2	86.4
*b* ()	157.7	108.2	89.6
*c* ()	86.4	118.2	133.1
()	90.0	90.0	90.0
()	113.0	90.0	101.4
()	90.0	90.0	90.0
Resolution range ()	43.792.66 (2.722.66)	39.402.80 (2.952.80)	31.212.40 (2.452.40)
Total No. of reflections	223707 (13525)	163919 (21497)	184649 (9636)
No. of unique reflections	61101 (4502)	28951 (3729)	75030 (4232)
Completeness (%)	99.9 (99.5)	93.2 (84.4)	96.5 (93.5)
Multiplicity	3.7 (3.0)	5.7 (5.8)	2.5 (2.3)
*I*/(*I*)	5.5 (1.0)	4.9 (1.2)	4.8 (1.4)
CC_1/2_	0.962 (0.390)	0.992 (0.717)	0.950 (0.491)
*R*_r.i.m._	0.272 (1.744)	0.260 (1.467)	0.239 (0.844)
Overall *B* factor from Wilson plot (^2^)	36.6	34.9	24.0
Refinement			
Resolution range ()	55.972.66 (2.722.66)	39.432.80 (2.872.80)	30.992.40 (2.462.40)
Completeness (%)	99.84 (98.23)	93.03 (83.43)	96.03 (93.02)
No. of reflections, working set	58196 (4236)	27454 (1785)	71230 (5062)
Final *R*_cryst_ (%)	18.7 (33.0)	25.5 (36.9)	19.1 (28.9)
Final *R*_free_ (%)	23.6 (38.7)	29.5 (45.1)	22.6 (33.0)
No. of non-H atoms			
Protein	14932	7512	14706
Ion/ligand	5	0	107
Water	11	0	594
Total	14948	7512	15407
R.m.s. deviations			
Bonds ()	0.015	0.009	0.013
Angles ()	1.640	0.981	1.506
Ramachandran plot			
Most favoured (%)	98.03	95.06	98.00
Allowed (%)	100.00	100.00	99.95
Outliers (%)	0.00	0.00	0.05
*MolProbity* score [percentile]	1.33 [100th]	1.17 [100th]	0.98 [100th]
Clashscore [percentile]	2.07 [100th]	6.1 [99th]	1.14 [100th]

**Table 2:** Peak heights and positions in native Patterson maps for CRMP-4 crystal form *B* data sets Patterson maps were calculated in the resolution range 104.

Data set	1	2	3	4	5
Resolution ()	2.8	2.9	3.0	3.1	3.2
First non-origin peak height (%)	71.0	59.9	65.6	62.4	64.7
First peak position (0.5, 0.5, *w*)	0.399	0.407	0.401	0.402	0.408
Second peak height (%)	33.4	24.4	27.5	32.1	29.0
Second peak position (0.0, 0.0, *w*)	0.201	0.186	0.197	0.195	0.184

**Table 3:** Comparison of refinement statistics for demodulation according to the lattice-alignment method (Wang, Kamtekar *et al.*, 2005 ▶) and DIGS ([Sec sec3.3]3.3) The same input model and comparable refinement parameters were used in both refinements using data set 1.

Demodulation method	No correction	Lattice alignment	DIGS
*R*_cryst_ (%)	31.1	27.7	26.5
*R*_free_ (%)	35.5	32.2	30.0
Map CC	63.9	66.9	67.1
Phase error ()	42.0	41.8	39.0

## Footnotes

1 Supporting information has been deposited in the IUCr electronic archive (Reference: DZ5326).
