# Supplementary material for: Crystal structure of human CRMP-4: correction of intensities for lattice-translocation disorder
Source: Acta Crystallogr D Biol Crystallogr. 2014 May 30;70(Pt 6):1680–94. doi: 10.1107/S1399004714006634 (PMC4051505; doi:10.1107/S1399004714006634)
Supplement: Supplementary file 1 [file d-70-01680-sup1.pdf]

Acta Crystallographica Section D

Volume 70 (2014)

Supporting information for article:

Crystal structure of human CRMP-4: correction of intensities for lattice-translocation disorder

Rajesh Ponnusamy, Andrey A. Lebedev, Steffen Pahlow and Bernhard Lohkamp

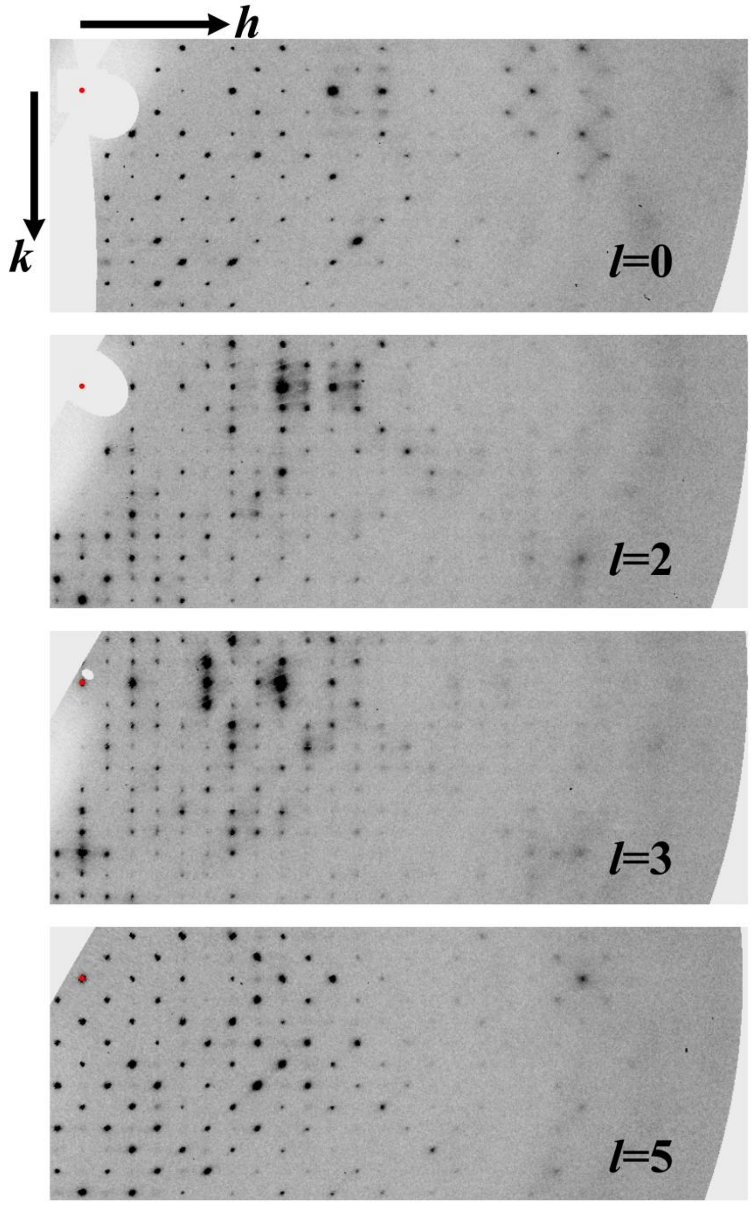

Figure S1 Pseudo-precession diffraction patterns from WT CRMP-4 crystals (form B) indicate 2-dimensional disorder. Shown are *hkl* planes for *l*=0, 2, 3, and 5. Layers for *l*=5n show sharp reflections. From the perpendicular directions the halos are seen as streaks, as in **Error! Reference source not found.**(a). The other reflections are diffuse in 2 dimensions with halos around the reflections. *LABELIT* (Sauter *et al.*, 2004) was used to calculate the diffraction image from experimental data and to produce the figure.

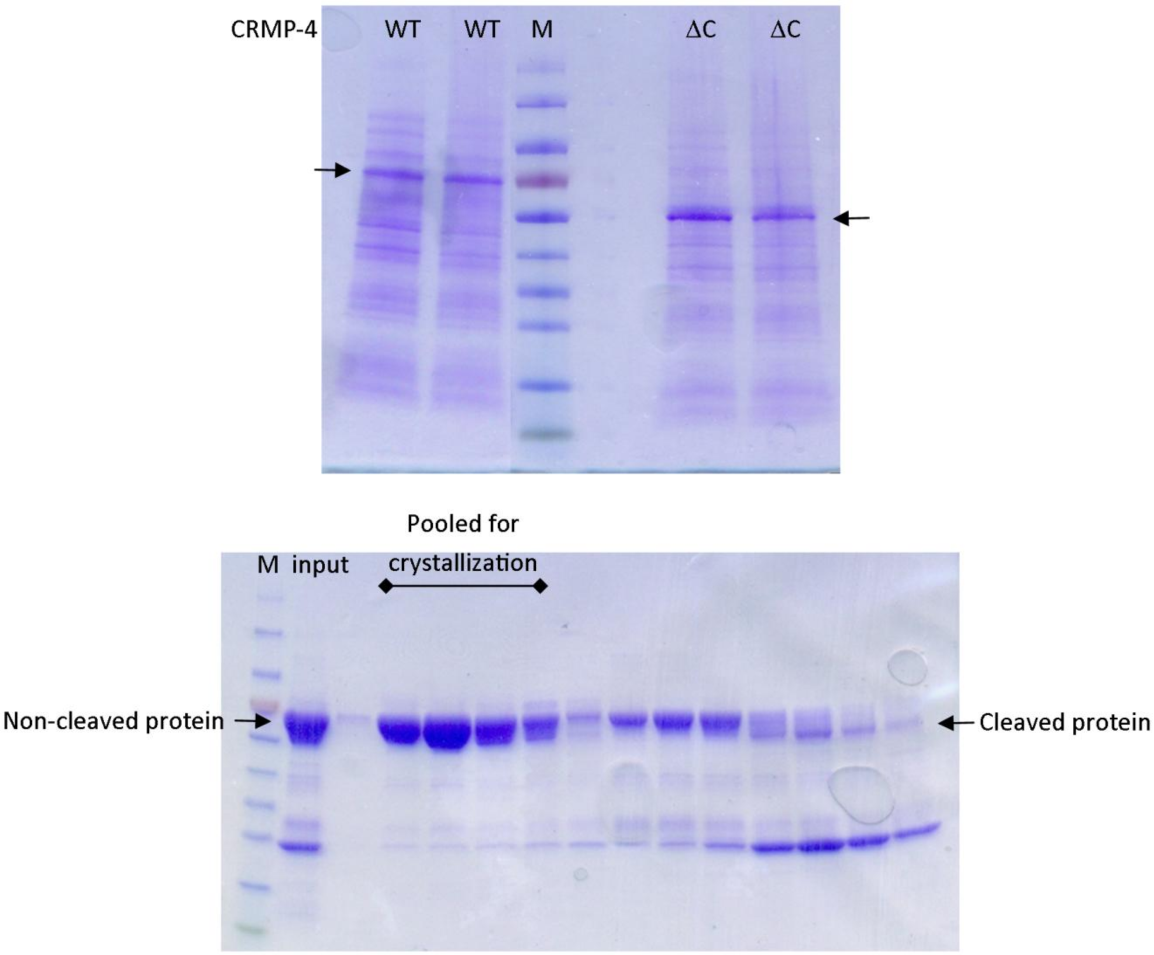

Figure S2 SDS-PAGE of CRMP-4 WT and ΔC. The top gel shows the expression levels of the two versions in the bacterial cell lysate. The bottom gel shows the protein content of fractions from size-exclusion chromatography to purify CRMP-4 WT for crystallisation.

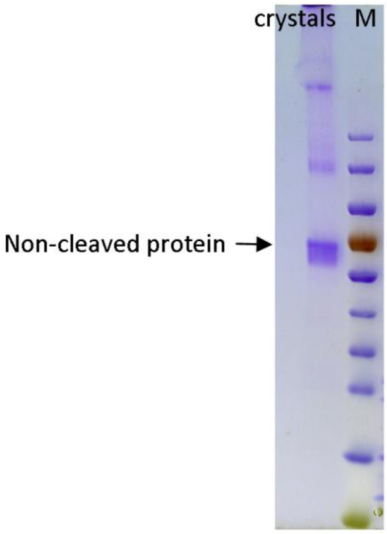

Figure S3 SDS-PAGE of CRMP-4 WT crystals. Approx. 15-20 crystals were washed extensively and dissolved in SDS running buffer before loading it onto the gel.

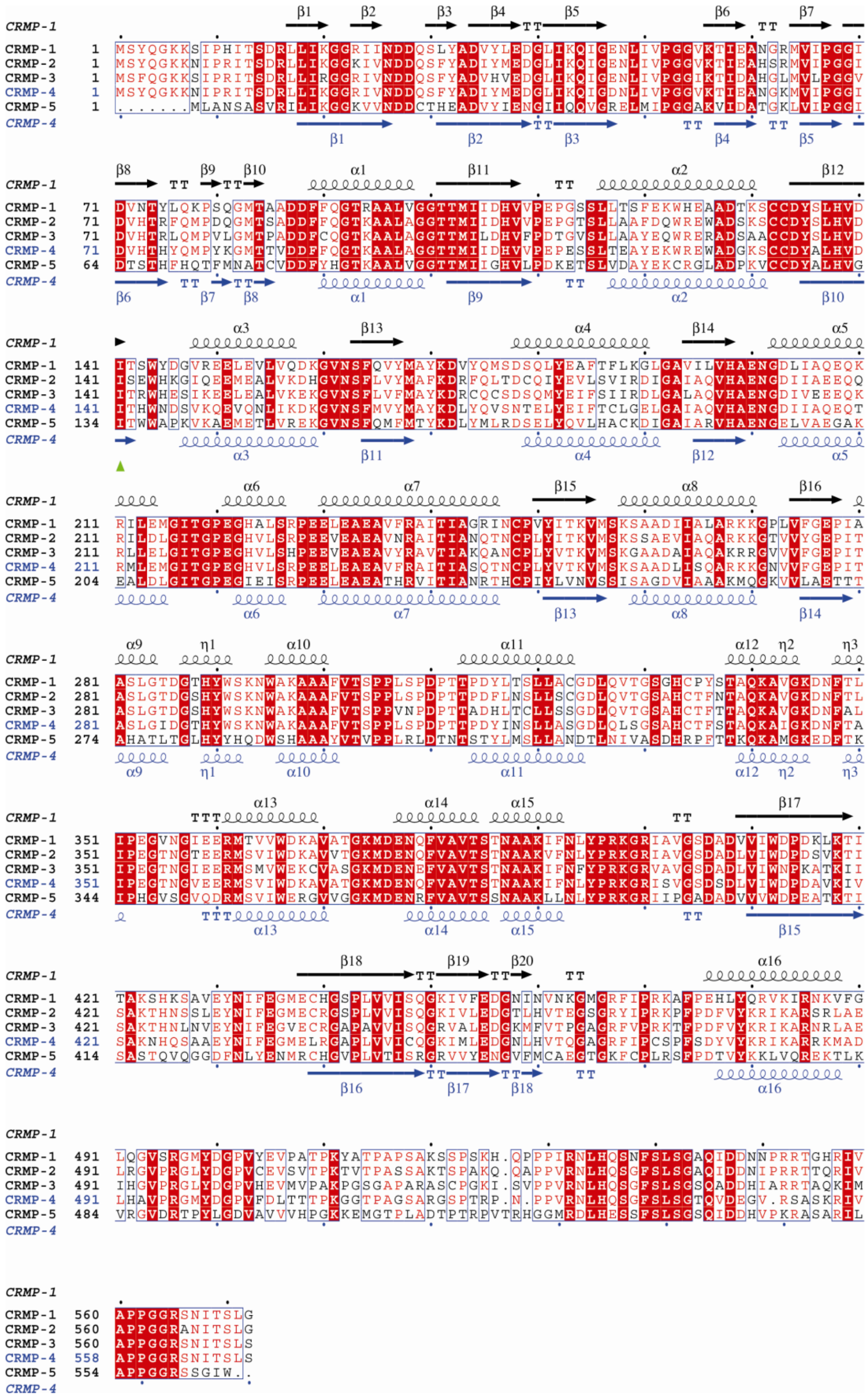

Figure S4 Sequence alignment of human CRMPs. The secondary structure elements for CRMP-1 (PDB code 1KCX) and CRMP-4 are shown above and below (in blue) the sequences, respectively. A green triangle indicates the mutation site I141V.

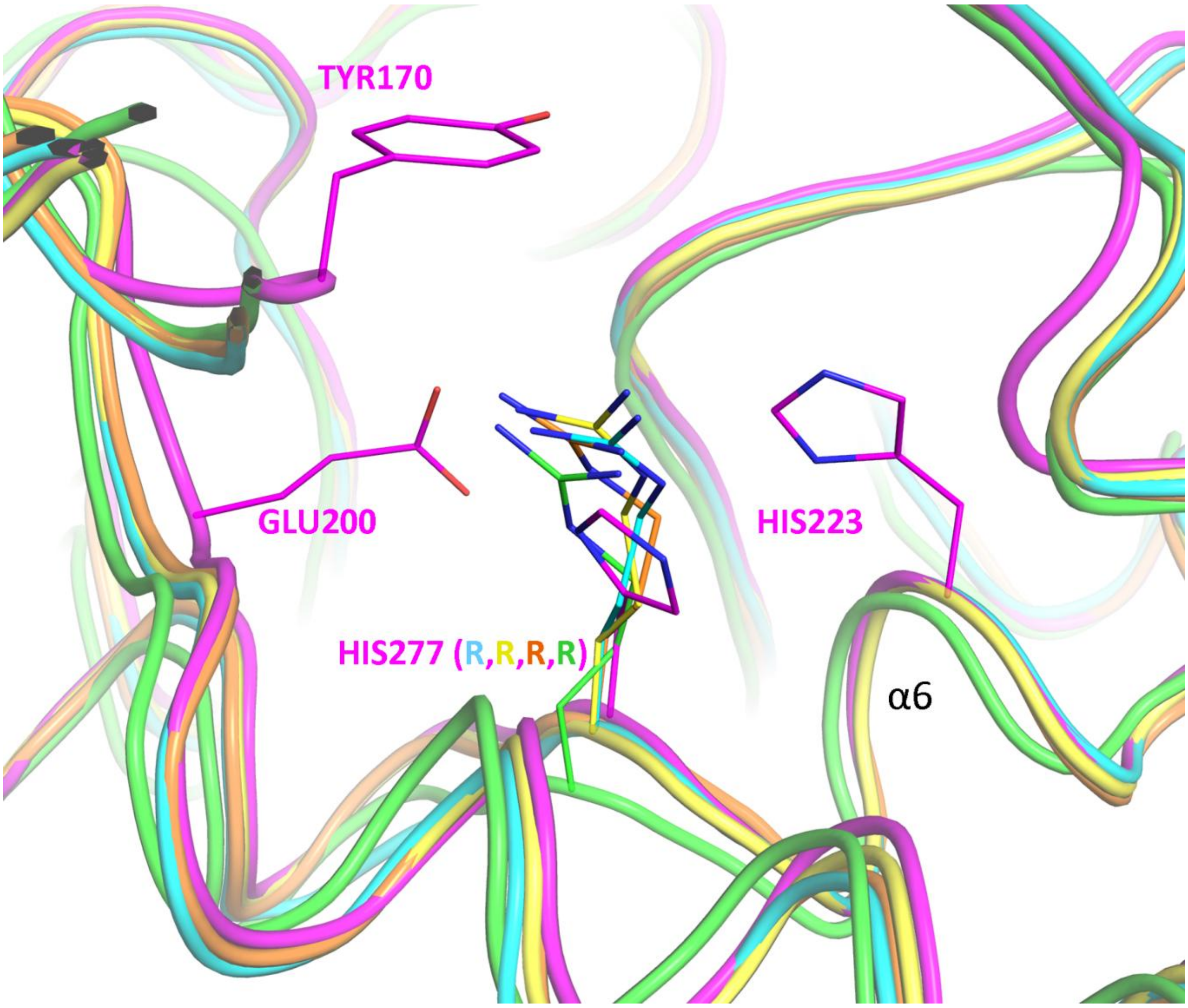

Figure S5 Putative active site in CRMP-3. CRMP-1 (cyan), CRMP-2 (yellow), CRMP-4 (orange) and CRMP-5 (green) are superimposed onto the model of CRMP-3 (magenta). Residues potentially involved in zinc ion binding and histone deacetylase activity are labelled and shown in stick.
